# *AIRE* Gene Mutation Presenting at Age 2 Years With Autoimmune Retinopathy and Steroid-Responsive Acute Liver Failure: A Case Report and Literature Review

**DOI:** 10.3389/fimmu.2021.687280

**Published:** 2021-05-28

**Authors:** Hirotaka Sakaguchi, Tatsuki Mizuochi, Masatoshi Haruta, Ryuta Takase, Shigeo Yoshida, Yushiro Yamashita, Ryuta Nishikomori

**Affiliations:** ^1^Department of Pediatrics and Child Health, Kurume University School of Medicine, Kurume, Japan; ^2^Department of Ophthalmology, Kurume University School of Medicine, Kurume, Japan

**Keywords:** *AIRE*, acute liver failure, autoimmune hepatitis, children, corticosteroid

## Abstract

Autoimmune polyendocrinopathy-candidiasis-ectodermal dystrophy (APECED) is a rare monogenic autosomal recessive disorder caused by mutation in the autoimmune regulator (AIRE) gene. Patients usually are diagnosed at ages between 5 and 15 years when they show 3 or more manifestations, most typically mucocutaneous candidiasis, Addison’s disease, and hypoparathyroidism. APECED-associated hepatitis (APAH) develops in only 10% to 40% of patients, with severity varying from subclinical chronic active hepatitis to potentially fatal acute liver failure (ALF). Ocular abnormalities are fairly common, most often keratopathy but sometimes retinopathy. Here we report a 2-year-old Japanese girl with an *AIRE* gene mutation who developed APAH with ALF, preceded by autoimmune retinopathy associated with anti-recoverin antibody before major symptoms suggested a diagnosis of APECED. Intravenous pulse methylprednisolone therapy followed by a corticosteroid combined with azathioprine treatment resolved ALF and achieved control of APAH. To our knowledge, our patient is the youngest reported to have ALF resulting from an *AIRE* gene mutation. Pulse methylprednisolone induction therapy followed by treatment with corticosteroid plus azathioprine may well be effective in other children with APAH and *AIRE* gene mutations.

## Introduction

Autoimmune polyendocrinopathy-candidiasis-ectodermal dystrophy (APECED; OMIM, 240300), also termed autoimmune polyendocrine syndrome type 1, is a rare monogenic autosomal recessive disease caused by mutations in the autoimmune regulator (AIRE) gene (1). APECED has been found to occur in approximately 1 in 90 000 to 200 000 individuals in most populations studied, mainly in European countries. The disease occurs more frequently in relatively isolated populations, being more prevalent among Iranian Jews (1:9000), Sardinians (1:14 000), Finns (1:25 000), and Slovenians (1:43 000). Norway (1:80 000), Poland (1:129 000), and France (1:500 000) show more typical frequencies of occurrence. In East Asia APECED is particularly infrequent; a rough estimate in Japan is 1:10 000 000 (2, 3).

AIRE participates in regulation of self-tolerance as T cells develop in the thymus. Defective function of AIRE encourages production of multiple anti-cytokine and organ-specific autoantibodies, leading to severe autoimmune disease that can damage multiple endocrine organs and other tissues. More than 20 different clinical manifestations have been associated with the disease; such high variability makes the course of disease unpredictable (4). Illness usually manifests during childhood, with additional clinical manifestations emerging later in life. APECED generally is diagnosed between the ages of 5 and 15 years (2). As a typical minimum, APECED patients manifest a triad of mucocutaneous candidiasis, Addison’s disease, and hypoparathyroidism. Patients may also exhibit autoimmune thyroiditis, Sjögren’s syndrome, premature ovarian/gonadal failure, anemia, diabetes mellitus, alopecia, vitiligo, gastritis, nail dystrophy, enamel hypoplasia, and hepatitis resembling autoimmune hepatitis (AIH), referred to as APECED-associated hepatitis (APAH) (5–7). APAH develops in 10% to 40% of patients with APECED, showing variable severity ranging from subclinical chronic active hepatitis to potentially fatal acute liver failure (ALF) (5–9). Ocular abnormalities are not infrequent; while keratopathy is most frequent, retinopathy may occur (1, 10–12). APECED is clinically defined based on the presence of at least 2 components of the classical triad; however, these 3 major criteria need not be met, especially when genetic testing demonstrates the mutation or minor criteria are met.

Here we report a 2-year-old Japanese girl with an *AIRE* gene mutation who developed ALF caused by APAH, as well as autoimmune retinopathy associated with anti-recoverin antibody that emerged before onset of major symptoms led to a diagnosis of APECED.

## Case Report

A previously healthy 2-year-old Japanese girl, the first child of healthy, non-consanguineous parents, presented with an episode of jaundice and vomiting. Growth and development were within the normal range, and no dysmorphic features were present. Hepatomegaly and jaundice were present. Neurologic findings were normal for age. Initial laboratory abnormalities included serum aspartate aminotransferase, 989 U/L (normal, <30); alanine aminotransferase, 1323 U/L (<30); gamma glutamyl transferase, 136 U/L (<40); total/direct bilirubin, 6.5/4.0 mg/dL (<1.2/<0.4); and immunoglobulin G, 2098 mg/dL (500 to 1280). Prothrombin time (INR) was 1.73 (0.75 to 1.15). Initial autoantibody screening, including antinuclear antibody (ANA), anti-smooth muscle antibody (SMA), and anti-liver kidney microsomal-1 antibody (LKM-1), was negative. Other causes of liver disease such as acute viral hepatitis and metabolic conditions were excluded by appropriate investigations. Abdominal magnetic resonance imaging (MRI) and ultrasonography showed a visible gallbladder and hepatomegaly; no choledochal cyst, bile duct dilation, tumor, or ascites was demonstrated. The patient was treated for ALF without hepatic encephalopathy, most likely caused by AIH. Intravenous pulse steroid therapy with methylprednisolone (pulse mPSL; 30 mg/kg/day for 3 days) was begun 2 days after admission. After pulse mPSL, we treated the patient with prednisolone (PSL, 1.5 mg/kg/day) and then added azathioprine (AZA; 1.2 to 2.0 mg/kg/day). As this combination therapy improved liver function, the PSL dose was tapered to 0.15 mg/kg/day (**Table 1**). After pulse mPSL had alleviated ALF, liver biopsy was performed. The specimen showed typical AIH findings including active interface hepatitis and predominance of plasma cells over lymphocytic inflammation. The patient was diagnosed with ALF as the cause of AIH; her score according to the system of the International Autoimmune Hepatitis Group was 20 points (13).

**Table 1:** Clinical course of laboratory results and treatment.

Laboratory value or treatment dose	Pretreatment	After 6 days	After 2 weeks	After 3 weeks	After 4 weeks	After 2 months	After 6 months
ALT (U/L; normal range, <30)	1323	455	175	318	151	41	22
GGT (U/L; <40)	136	200	239	345	194	29	12
T Bil (mg/dL; <1.2)	6.5	3.4	1.5	1.4	0.9	0.6	0.7
D Bil (mg/dL; <0.4)	4.0	1.9	0.4	0.5	0.1	0.1	0.1
PT-INR (0.75 to 1.15)	1.73	1.15	0.96	0.98	0.9	0.99	n.d.
IgG (mg/dL; 500 to 1280)	2098	n.d.	1079	n.d.	n.d.	970	1170
mPSL (mg/kg/day)	30 × 3 days						
PSL (mg/kg/day)		1.5	1.5	1.2	1.2	0.4	0.15
AZA (mg/kg/day)		-	-	-	1.2	1.2	2.0

ALT, alanine aminotransferase; GGT, gamma glutamyl-transferase; T Bil, total bilirubin; D Bil, direct bilirubin; PT-INR, prothrombin time-international normalized ratio; n.d., not done; IgG, immunoglobulin G; mPSL, methylprednisolone; PSL, prednisolone; AZA, azathioprine; -, no treatment.

**Figure 1:**
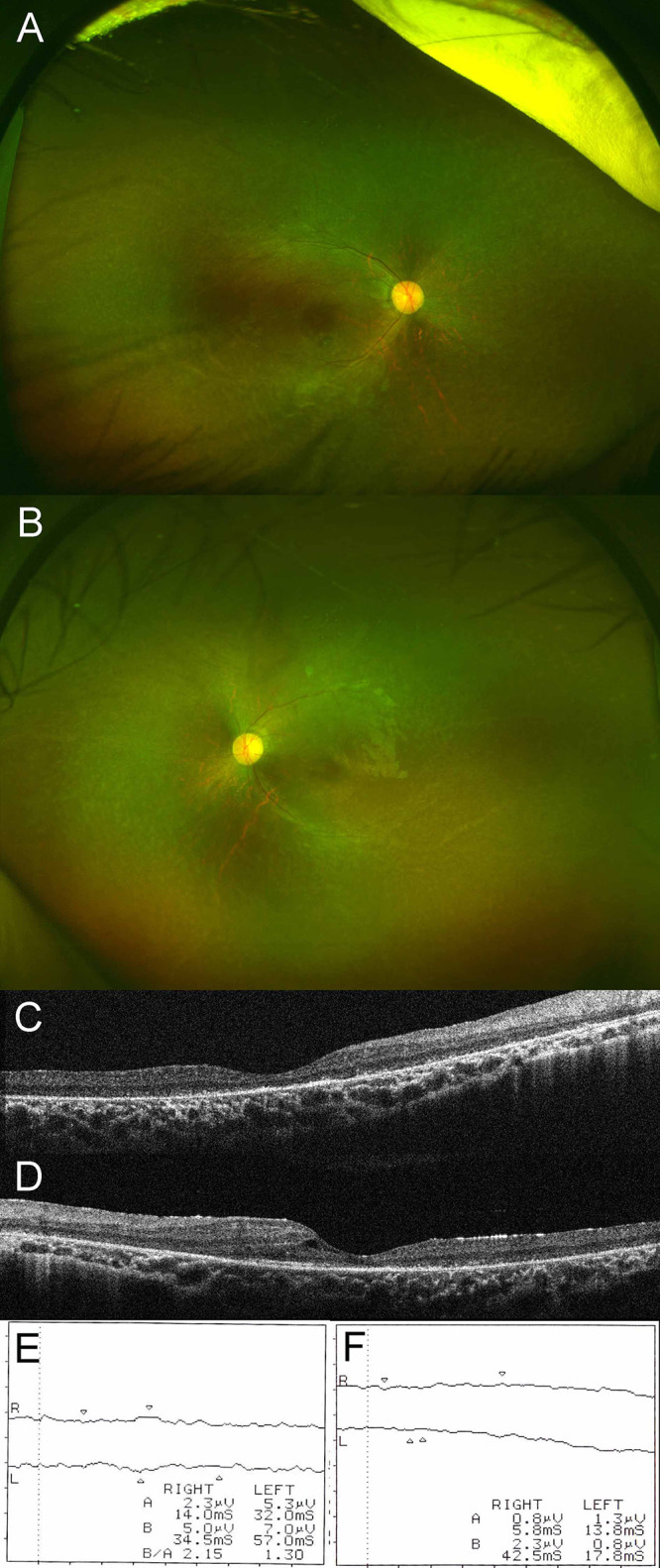
Ultra-widefield fundus photograph of right **(A)** and left **(B)** eyes. Dilated fundus examination disclosed vascular attenuation, optic disc pallor, and retinal pigmentary changes in both eyes. Macular optical coherence tomography of right **(C)** and left **(D)** eyes. Macular optical coherence tomography demonstrated marked disruption of the external limiting membrane and an ellipsoid zone in both eyes, with small foveal cysts in the left eye. Results of scotopic **(E)** and photopic **(F)** electroretinograms. Scotopic and photopic electroretinograms showed no recordable responses in either eye.

The patient was referred to an ophthalmologist for evaluation of possible adverse effects of long-term systemic corticosteroid treatment. Best-corrected visual acuity was no light perception in the right eye and 5/20 in the left eye, with eccentric fixation and exotropia in the right eye. Anterior slit-lamp examination had no remarkable findings. A dilated fundus examination showed vascular attenuation, optic disc pallor, and retinal pigmentary changes in both eyes (**Figures 1A, B**). Macular optical coherence tomography (OCT) demonstrated marked disruption of the external limiting membrane with an ellipsoid zone in both eyes, and small foveal cysts in the left eye (**Figures 1C, D**). Scotopic and photopic electroretinograms showed no recordable responses in either eye (**Figures 1E, F**). Ultra-widefield fundus autofluorescence examination showed a subtle ring of excessive autofluorescence in the parafoveal regions in both eyes, and ultra-widefield OCT demonstrated diffuse thinning of both retinas with loss of outer retinal structures, as reported in a previous diagnostic image presentation (12). Serologic tests were positive for anti-recoverin antibody and negative for anti-alpha enolase antibody. Based on these ocular and serologic findings, the patient was additionally diagnosed with bilateral autoimmune retinopathy. Systemic evaluation including MRI, ultrasonography, and serum tumor marker determinations such as alpha-fetoprotein, carbohydrate antigen 19-9, carcinoembryonic antigen, and squamous cell carcinoma-related antigen disclosed no underlying malignancy at the age of 3 years.

Since the patient had both AIH and autoimmune retinopathy, we suspected an inborn error of immunity. We therefore performed a 400-gene panel analysis for such inborn errors in the patient using next-generation sequencing, which identified a homozygous disease-causing variant in the *AIRE* gene (c.415C>T, p.139*). Each parent was found to be heterozygous for the variant. Additionally, the patient’s 1-year-old sister, who had been treated at another hospital for encephalopathy of unknown cause, was genetically tested and found to be homozygous for the same variant.

At this writing the patient was 3 years and 11 months old, and had no manifestations of APECED other than APAH and autoimmune retinopathy. Additionally, test results for serum autoantibodies typical of AIH such as ANA, SMA, and LKM-1 all were negative at that time.

## Discussion

Here we report a Japanese girl with *AIRE* gene mutation who presented at 2 years of age with ALF caused by APAH. The patient also was found to have an autoimmune retinopathy associated with anti-recoverin antibody. Pulse mPSL therapy followed by corticosteroid plus AZA treatment brought about recovery from ALF and control of APAH, respectively. The patient did not present with any of the conditions representing the classical clinical triad of APECED (mucocutaneous candidiasis, Addison’s disease, and hypoparathyroidism). In rare instances, other manifestations such as APAH and autoimmune retinopathy may precede the classical clinical symptoms as the presenting features of APECED.

APAH, which develops in 10% to 40% of patients with APECED, has been reported to cause ALF in some APECED cases (5–9). In a series of 68 patients with APECED from Finland, 12 had APAH including 2 who died of ALF at 7 and 17 years of age (5), while among 41 patients from Italy, 8 had APAH including 1 who died of ALF at 11 years of age (6). To our knowledge, our APECED patient is the youngest, at 2 years, to develop ALF caused by APAH. In **Table 2**, we summarize clinical, genetic, and treatment aspects of APAH patients with *AIRE* gene mutation in a reported case series (7) and in our patient. Compared with more common forms of AIH, APAH is characterized by an earlier onset of hepatitis and a lower positive rate for AIH-associated serum autoantibodies such as ANA, SMA, and LKM-1, as well as a more favorable response to immunomodulatory treatment. Available published information seems incomplete and inconclusive regarding treatment and outcome of patients with APAH. However, Zachou et al. (14) reported that high-dose intravenous corticosteroid was safe and effective for treatment of patients with acute severe AIH, while Sogo et al. (15) advocated pulse mPSL as a safe and effective therapy for children with AIH. Conventional treatment of childhood AIH consists of prednisolone at 2 mg/kg/day, to be gradually tapered over 4 to 8 weeks guided by declines in serum transaminases until a maintenance dose of 2.5 to 5 mg/day is reached (16). In general, AZA is added as a steroid-sparing agent when transaminase concentrations no longer respond to prednisolone alone or, rarely, when prednisolone produces serious adverse effects. AZA or 6-mercaptopurine with or without corticosteroids has been used in most APAH patients. Among the APAH patients in one report, 89% responded to the initial immunomodulatory regimen used to control hepatitis (7). In our present case, pulse mPSL was effective against ALF, and a combination of PSL and AZA provided effective induction and maintenance treatment in APAH. We would encourage a similar approach in APAH patients with ALF and an *AIRE* gene mutation.

**Table 2:** Clinical, genetic, and treatment aspects of APAH patients with *AIRE* gene mutation in a reported series and in our patient.

Reference, year, and number of patients	Median age at diagnosis of hepatitis, years (range)	*AIRE* gene mutation (percentage of patients with indicated mutation)	Percentage of patients positive for serum autoantibody			Maintenance treatment (percentage of patients)	Response to immunomodulatory treatment
			ANA	SMA	LKM-1		
Chascsa et al., 2021 (7) 18 patients	7 (2 to 31)	c.967_979del13 (72%) c.769C>T (17%)	11%	0%	0%	PSL+AZA (33%) AZA or 6MP monotherapy (22%) PSL monotherapy (11%) MMF+rituximab (11%) CS monotherapy (6%) MMF monotherapy (6%) PSL+MMF+TAC (6%) 6MP+CS (6%)	All 18 patients with APAH had their disease in clinical and biochemical remission
Our patient	2	c.415C>T	Negative	Negative	Negative	PSL+AZA	Clinical and biochemical remission

APAH, APECED-associated hepatitis; AIRE, autoimmune regulator; ANA, antinuclear antibody; SMA, anti-smooth muscle antibody; LKM-1, anti-liver kidney microsomal-1 antibody; PSL, prednisone or prednisolone; AZA, azathioprine; 6MP, 6-mercaptopurine; MMF, mycophenolate mofetil; CS, cyclosporine; TAC, tacrolimus.

Lankisch et al. (17) reported that among 25 children with AIH, *AIRE* gene analysis detected a heterozygous mutation in 4 patients, so even a heterozygous *AIRE* mutation might predispose to childhood AIH. We believe that the possibility of *AIRE* gene mutations should be investigated in children with early- onset AIH (e.g. under 6 years of age); if a pathogenic mutation is identified, one should treat the patient with a combination of corticosteroid and AZA-preceded by pulse mPSL if ALF is present.

Ocular complications previously reported in patients with APECED include autoimmune keratopathy and retinopathy (1, 10–12). Autoantibodies directed against lacrimal gland, cornea, or retina are believed to be responsible for the ocular manifestations of APECED. Bourgault et al. (11) reported 4 patients diagnosed with APECED who were found to have bilateral retinopathy and antiretinal antibodies. In addition to APAH, our 2-year-old patient with genetically confirmed APECED developed bilateral autoimmune retinopathy. She also appears to be among the youngest patients described as positive for anti-recoverin antibody (11). Although anti-recoverin antibody has been considered highly correlated with cancer (18, 19), systemic evaluation has disclosed no underlying malignancy in the present patient. Autoimmune retinopathy should be considered in the differential diagnosis of unusual retinal degeneration in patients with *AIRE* mutations, and prompt systemic work-up is needed to rule out occult malignancy in those positive for anti-recoverin antibody. APECED-associated retinal complications recently have drawn attention as a relatively common complication of this disorder; however, no effective treatment for APECED-associated retinopathy has been identified (10). Couturier et al. concluded that no treatment has shown efficacy against APECED-associated retinopathy (10). Bourgault et al. reported 3 patients who received different immunosuppressants during various stages of APECED-associated retinopathy; in all 3, retinopathy, vision, and/or electroretinographic findings continued to worsen despite immunosuppressive treatment (11).

Prevalence data for disease manifestations among patients with APECED in various countries and regions including Finland, Italy, Norway, Russia, North and South America, and Japan are summarized in **Table 3** (5, 6, 9, 20–22). Among Japanese patients with APECED, Addison’s disease, hypoparathyroidism, autoimmune thyroiditis, nail dystrophy, and enamel hypoplasia are less prevalent than in patients from other countries, while type 1 diabetes is more prevalent.

**Table 3:** Prevalence of disease manifestations in patients with APECED from various countries and regions.

	Frequency (%)					
	Japan ^20)^	Finland ^5)^	Italy ^6)^	Norway ^21)^	Russia ^22)^	North and South America ^9)^
N	10 *	68	41	36	46	35 ^#^
Classic triad						
Candidiasis	90	100	83	79	70	86
Addison’s disease	40	72	73	68	54	83
Hypoparathyroidism	60	79	93	82	83	91
Other manifestations						
Hepatitis	40	12	20	3	11	43
Ocular manifestations	10	35	12	9	7	29
Autoimmune thyroiditis	0	4	10	9	20	23
Type 1 diabetes	30	12	2	9	11	11
Nail dystrophy	0	52	7	14	N/A	17
Enamel hypoplasia	10	77	N/A	37	N/A	86

APECED, autoimmune polyendocrinopathy-candidiasis-ectodermal dystrophy; N, number of patients; N/A, not available.

*The Japanese patients include our own, whom we added to the 9 Japanese patients in ref. 20 for these frequency calculations.

^#^Subjects from the Americas include 33 patients from the United States, 1 from Canada, and 1 from Colombia.

In conclusion, we believe that our patient is the youngest reported to date to have ALF caused by an *AIRE* mutation. Pulse mPSL induction therapy followed by corticosteroid plus AZA was effective against ALF and APAH.

## Data Availability Statement

The raw data supporting the conclusions of this article will be made available by the authors, without undue reservation.

## Ethics Statement

The studies involving human participants were reviewed and approved by Ethical Committee of Kurume University. Written informed consent to participate in this study was provided by the participants’ legal guardian/next of kin, for the publication of any potentially identifiable images or data included in this article.

## Author Contributions

HS and TM conceptualized and designed the study, collected data, drafted the initial manuscript, and reviewed and revised the manuscript. MH collected data, drafted the initial manuscript, and reviewed and revised the manuscript. RT performed genetic analysis and reviewed and revised the manuscript. SY collected data and reviewed and revised the manuscript. YY collected data and reviewed and revised the manuscript. RN performed genetic analysis, drafted the initial manuscript, and reviewed and revised the manuscript. All authors contributed to the article and approved the submitted version.

## Conflict of Interest

The authors declare that the research was conducted in the absence of any commercial or financial relationships that could be construed as a potential conflict of interest.

## Abbreviations

AIH, autoimmune hepatitis; AIRE, autoimmune regulator; ALF, acute liver failure; ANA, antinuclear antibody; APAH, APECED-associated hepatitis; APECED, autoimmune polyendocrinopathy-candidiasis-ectodermal dystrophy; AZA, azathioprine; LKM-1, anti-liver kidney microsomal-1 antibody; mPSL, methylprednisolone; MRI, magnetic resonance imaging; OCT, optical coherence tomography; PSL, prednisolone; SMA, anti-smooth muscle antibody.
